# Notes Respecting Special Cathartics

**Published:** 1887-06

**Authors:** 


### *NOTES RESPECTING SPECIAL CATHARTICS.*

Dr. Henry M. Field (*Journal of the American Medical Association*) calls attention to the following points respecting a few cathartics:

1. The salines do not agree with the aged—they find them too chilling; and a dose of epsom salts, which may operate very kindly upon the young and middle-aged and vigorous, may bring a serious disaster to the old man or woman. A sudden depression of vital energy and the function of calorification thus procured, together with other favoring circumstances, have more than once precipitated the subject into fatal pneumonia.

2. All cathartics are apt to be attended with colicky complications when given to a woman at the epoch of menopause; and especial combination at such time, as with carminatives, should be directed against this painful action.

3. The common domestic cathartic, senna, should never be prescribed to the subject of cumulative constipation or of impacted feces; if there be anything answerable to a fæcal plug formed in the course of the small intestine or near the valve, on either side, such a peristaltic ca'hartic as senna will infallibly occasion serious, and even alarming, colic before evacuation can be accomplished; and the same restriction applies to a similar use of an integral dose of calomel.

4. In a case of impacted constipation, in which it is presumed that the bowels are more or less distended with hard, dry, knotty, scybalous masses, nothing works so well as epsom salts, combined, perhaps, with small doses of tartar emetic.

5. In cases of uterine hæmorrhage, habitual constipation can best be treated by cream of tartar. This does not induce muscular contraction of either the intestines or uterus, while it reduces temperature and lowers blood pressure.

